# Association

**Published:** 1886-02

**Authors:** 


### Association.

We recently enjoyed the privilege of attending the regular monthly meeting of the Detroit Dental Society. It was organized several years ago and has been well sustained from the beginning, indeed has been growing all the time, and has been the means of great good to the profession in that city. No pretensions have been made in the way of elaborate work, or special demonstrations, but the work has been effective upon those for whom it is intended, as is shown by the fact that entire harmony and fraternity prevails in the profession there, and the knowledge and information of each one becomes common property to all.

The permanency and success of the Society is still further established by the very liberal act of Mr. Calkins, the dealer in dental supplies, he having provided an excellent room, comfortably and beautifully furnished for the use of the Society, without any charge. This Society may well be regarded as a model city society, such as should exist and be well sustained in every city in our country of twenty thousand inhabitants and over; quite a number have such bodies, but a good many have not. And why not? Simply because they will not. We call upon all such to be up and doing.

THE Fourth Annual Commencement Exercises of the College of Dentistry of the University of California, were held in connection with those of the medical department at the Grand Opera House, San Francisco, Tuesday, November, 10th, at 2 o'clock P.M.

ORDER OF EXERCISES.

PRAYER.—Rev. T. K. Noble.

ADDRESS ON BEHALF OF THE MEDICAL DEPARTMENT.—Professor Benj. R. Ewan, M. D.

ADDRESS ON BEHALF OF THE DENTAL DEPARTMENT.—Professor Maurice J. Sullivan, D. D. S.

CONFERRING DEGREES OF DOCTOR OF MEDICINE.—CONFERRING DEGREES OF DOCTOR OF DENTAL SURGERY.

ADMINISTRATION OF THE HIPPOCRATIC OATH TO THE GRADUATES OF MEDICINE.—Professor R. Beverly Cole, A. M., M. D., M. R. C. S., Eng. Chairman of the Faculty of Medicine.

BENEDICTION.—Rev. T. K. Noble.

Appropriate music was provided by the orchestra.

The number of matriculates for the session was thirty-seven.

The Degree of Doctor of Dental Surgery was conferred upon the following graduates:

Harry Sylvester Bettis,

George Botsford,

Daniel Barratt Cate,

Nathanial Thomas Coulson,

George Ihnier Drucker,

William Ellis Fitzpatrick,

Walter Robert Henderson,

John Adams Douglass Hutton,

Franklin Pancoast,

Charles Theodore Rodolph,

Frederick Judson Saxe, A. M.

Joseph Schneider,

Henry Sylvester. Jr.

